# ClickIn: a flexible protocol for quantifying mitochondrial uptake of nucleobase derivatives

**DOI:** 10.1098/rsfs.2016.0117

**Published:** 2017-04-06

**Authors:** Kurt Hoogewijs, Andrew M. James, Robin A. J. Smith, Frank Abendroth, Michael J. Gait, Michael P. Murphy, Robert N. Lightowlers

**Affiliations:** 1 Medical Research Council Laboratory of Molecular Biology, Cambridge, UK; 2 Medical Research Council Mitochondrial Biology Unit, Cambridge, UK; 3 Department of Chemistry, University of Otago, Dunedin, New Zealand; 4 The Wellcome Trust Centre for Mitochondrial Research, Institute for Cell and Molecular Biosciences, The Medical School, Newcastle University, Newcastle upon Tyne, UK

**Keywords:** click chemistry, mitochondria, nucleobase derivatives, mitochondrial disease

## Abstract

There is an increasing interest in targeting molecules to the mitochondrial matrix. Many proteins are naturally imported through the translocase complexes found in the outer and inner mitochondrial membranes. One possible means for importing molecules is therefore to use a mitochondrial pre-protein as a vector and assess what forms of molecules can be attached to the pre-protein as cargo. A major difficulty with this approach is to ensure that any chimaeric molecule does indeed access the mitochondrial matrix and does not merely associate with the mitochondrial membranes. We have recently demonstrated that click chemistry can be used both to demonstrate convincingly mitochondrial import of a peptide–peptide nucleic acid conjugate and also to quantify the mitochondrial uptake for specific synthetic conjugates. We now report an adaptation of the synthesis to facilitate simple quantification of multiple molecules and hence to calculate the efficiency of their mitochondrial import.

## 1. Introduction

Human mitochondrial DNA (mtDNA) is found in large copy number in nearly all nucleated cells. In mammals, this small (16 569 bp) genome encodes just 13 polypeptides and all the RNA components (2 mt-rRNAs and 22 mt-tRNAs) required for their intra-mitochondrial synthesis. All 13 proteins are members of four of the five multi-subunit complexes of the mitochondrial inner membrane that couple oxidative phosphorylation (OXPHOS). Mutations in the mitochondrial genome can cause a wide variety of progressive disorders mostly affecting the muscle or nervous system [1] and often the patient will present with two subpopulations of mtDNA, one wild-type and one mutated, a situation termed heteroplasmy [2]. Mutations would be predicted to affect the levels of normal mtDNA-encoded proteins, either directly by resulting in the loss or mutation of protein-coding genes, or by a similar loss or mutation of mt-RNA affecting the synthesis of these proteins. Thus, mutations would be predicted to cause a defect in the maintenance of the OXPHOS machinery. Pathogenesis, however, is complicated by heteroplasmy. As mutations are nearly always recessive, cells can tolerate the presence of a level of mutated mtDNA without any loss of OXPHOS activity. Indeed, OXPHOS dysfunction only becomes apparent when mutation loads rise above a certain threshold level, a level that is reported to vary dependent on the pathogenic mtDNA mutation and the cell type [3]. To date, there is no effective treatment for the vast majority of patients with mtDNA disease.

Over 20 years ago, we suggested that it might be possible to try and manipulate the level of heteroplasmy as a potential therapeutic intervention [4]. We reasoned that if it were possible to selectively target the mutated mtDNA and prevent it from replicating, the wild-type would propagate over time, potentially restoring OXPHOS and slowing progression of the disorder. This antigenomic approach to treating patients with mtDNA disease requires the design of an antigenomic agent that can target mitochondria in whole cells and be imported into the mitochondrial matrix where the mtDNA resides. To facilitate selective binding to the mutated molecule, nucleobase derivatives such as peptide nucleic acids (PNAs) were used [5]. Novel synthetic derivatives were targeted to mitochondria as a function of their charge, and were predicted to promote the direct transfer of chimaeric molecules across membranes. Unfortunately, although many candidates have been synthesized, and several methods have demonstrated a clear co-localization of the antigenomic molecules to mitochondria in whole cells, resolution of these techniques has never been sufficient to convincingly show that the molecules have accessed the mitochondrial matrix [6]. A second approach was to add a synthetic mitochondrial pre-protein to the derivatives, to exploit the natural protein import machinery of the mitochondrion. The mitochondrial processing peptidase (MPP) would be predicted to cleave the mitochondria targeting sequence (MTS) of imported proteins, and thus, by extension, peptide–PNA conjugates [7]. While cleavage of the MTS by MPP is often regarded as evidence of protein uptake into isolated mitochondria, it has been shown that cleavage of the MTS can also occur to some extent while the cargo is still located bound to the translocase at the inner membrane, resulting in erroneous interpretation of the results [8].

**Figure 1.**
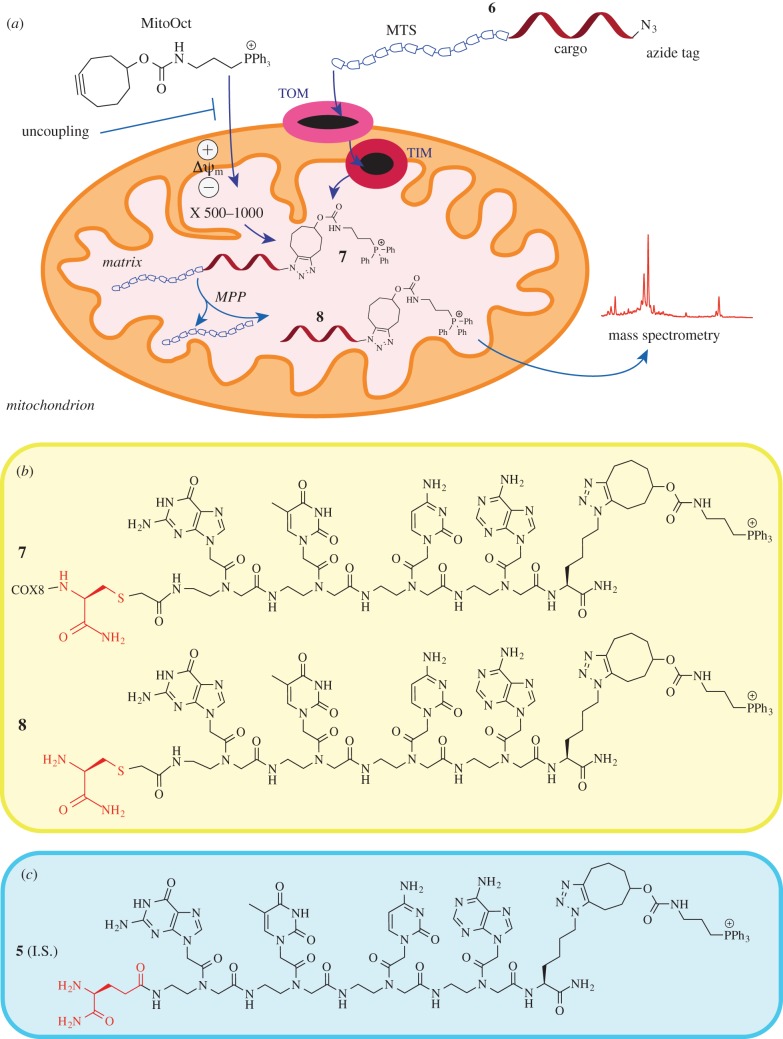
(*a*) The ClickIn strategy makes use of a slow but bioorthogonal reaction between MitoOct and an azide-labelled peptide–PNA conjugate **6.** Upon click reaction and import, the MTS is cleaved from the clicked conjugate **7** presumably by the MPP and the resulting product **8** is quantified using MALDI-TOF MS as described. (*b*) Structures of the clicked conjugate **7** and its MPP processed form **8**. (*c*) To quantify the formed clicked PNA **8**, a suitable internal standard **5** was synthesized using isoglutamine to replace the Cys(Ac)-NH_2_. All syntheses are described in the Experimental procedure section. (Online version in colour.)

Recently, we have used the ClickIn strategy to show unequivocally that a mitochondrial pre-protein carrying a short peptide nucleic acid as cargo could be imported through the natural mitochondrial protein import machinery [9]. To do this, we used the mitochondrial membrane potential to concentrate the mitochondrially targeted cyclooctyne, MitoOct [10] and measured the formation of the intact clicked conjugate on import of a peptide–PNA–azide (figure 1). With the appropriate negative controls, this technique can easily confirm the localization of compounds in the mitochondrial matrix. In this report, we present a modified ClickIn strategy, where we make use of both the ClickIn reaction in energized mitochondria and the removal of the MTS by MPP. The combination of both strategies allows both qualitative and quantitative observation of mitochondrial uptake. Furthermore, we report an optimized method for the synthesis of the required peptide–PNA conjugates. The synthesis of such large molecules (more than 30 residues) in a linear manner can be challenging, with side products resulting from incomplete steps in the synthesis as well as the final cleavage and deprotection step contaminating the product. To resolve this issue, a simple strategy for ligation of independently synthesized peptide and PNA moieties has been designed. The conjugation of peptides and PNAs using cysteine alkylation has been previously described and is well established in the field [11,12]. Traditionally, this reaction has proved to be highly reliable, with few side products caused by alkylation of other nucleophilic residues (e.g. amines or imidazoles). In addition, the reaction is carried out under very mild conditions at neutral to slightly basic pH (7.5–8) in an aqueous buffer, avoiding the possibility of epimerization of the amino acids in the peptide.

## 2. Experimental procedure

### 2.1. Materials

**Figure 2.**
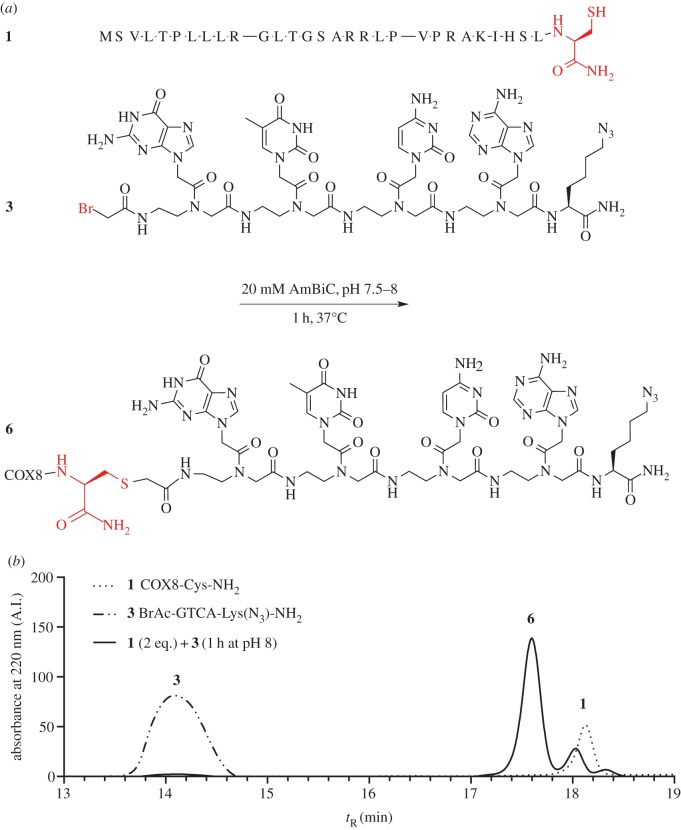
(*a*) Scheme showing the reaction between cysteine modified COX8 peptide **1** and bromo-acetylated PNA **3**. (*b*) Overlay of chromatograms of **1**, **3**, and the crude reaction mixture containing **6** after 1 h at 37°C are shown. All syntheses are described in the Experimental procedure section. (Online version in colour.)

Fmoc-PNA monomers were obtained from Link Technologies (UK). *N*-alpha-*t*-butyloxycarbonyl-l-glutamic amide (Boc-l-Glu-NH_2_) was obtained from Iris Biotech (Germany). Fmoc protected amino acids were obtained from AGTC Bioproducts (UK). All other reagents and chemicals were obtained from Sigma-Aldrich. Mass spectrometry was performed on an Applied Biosystems Voyager-DE PRO MALDI-TOF spectrometer in linear mode, and processed using mMass. HPLC purification was performed on a Varian 940-LC equipped with a Phenomenex Luna C18 column (250 × 10 mm, 10 µm) using a flow rate of 4 ml min^−1^. For the chromatograms in figure 2 a gradient of 0–75% B in 25 min was used (A is 0.1% TFA in H_2_O, B is 0.1% in acetonitrile). HPLC chromatograms of purified compounds are provided in the electronic supplementary material.

### 2.2. Peptide synthesis and deprotection

Peptides and PNAs were synthesized on a Liberty Blue peptide synthesizer using microwave irradiation on a Rink amide ChemMatrix support using Fmoc chemistry with five equivalents of Fmoc-amino acids, *N*,*N*′-diisopropyl-carbodiimide (DIC) and ethyl (hydroxyimino)cyanoacetate (Oxyma) in a 1 : 1 : 1 ratio in dimethylformamide (DMF) unless otherwise specified.

After synthesis, the resin was washed with DMF (3×), dichloromethane (3×) and diethyl ether (3×) and dried *in vacuo* for a minimum of 3 h. The peptides and PNAs were cleaved off the resin and deprotected by addition of an acidic cleavage cocktail for 60–90 min. Then, the solution was filtered off the resin, followed by washing of the resin (3 × 2 ml). The solution was evaporated using nitrogen flow, reducing the volume to 2 ml, after which 10 ml of cold diethyl ether was added, precipitating the peptide. The suspension was centrifuged and the supernatant was decanted. The pellet was resuspended in 10 ml of cold diethyl ether, followed by centrifugation, the ether was removed by decantation and the pellet dried *in vacuo*. The dried peptide was redissolved in 1 ml of Milli-Q water and purified on HPLC, where fractions with purity more than 95% (by MS) were selected.

### 2.3. COX8-Cys-NH_2_ (**1**)

The peptide was synthesized on a 50 µmol scale. Fmoc-Cys(Trt)-OH and Fmoc-His(Trt)-OH were coupled at 50°C, other amino acids were coupled at 75°C for 10 min. Fmoc-Arg(Pbf)-OH was coupled using double couplings, as well as residues 1 to 12 (N-terminus). In total, 10 µmol of the peptide was deprotected and cleaved off the resin using 5 ml of TFA/DODT/TIS/H_2_O (94 : 2,5 : 1 : 2,5) for 90 min. Purification of the peptide on HPLC was carried out using a 0–100% gradient (3 min 100% A, then to 100% B in 22 min, with the peptide eluting at 65%B): *m*/*z* 3255.64 observed (3254.91 calculated).

### 2.4. GTCA-Lys-(N_3_)-Rink amide ChemMatrix (**2**)

In total, 25 µmol resin was allowed to swell in DMF for 10 min, after which 75 µmol Fmoc-azido-lysine (Fmoc-Lys(N_3_)-OH), 75 µmol benzotriazole-1-yl-oxy-tris-pyrrolidino-phosphonium hexafluorophosphate and 150 µmol *N*,*N*-diisopropylethylamine dissolved in DMF was added to the resin. The resin was shaken for 90 min, after which the reaction mixture was filtered and the resin was washed with DMF (6 × 2 ml). The resin was loaded into the Peptide Synthesizer and the PNA was synthesized by automated peptide synthesis. Couplings of 30 min at 75°C were followed by a capping step (5% acetic anhydride, 6% 2,6-lutidine in DMF) for 5 min at room temperature. Fmoc-deprotection was carried out in two treatments with 20% piperidine in DMF for 1 and 3 min at room temperature. The resin, now with the completed sequence, was washed with DMF, DCM and diethyl ether (3× each) and dried *in vacuo*.

### 2.5. Bromo-Ac-GTCA-Lys-(N_3_)-NH_2_ (**3**)

In total, 20 µmol **2** was left to swell in DMF for 30 min. In a separate tube, 200 µmol DIC was mixed with 200 µmol bromoacetic acid in 400 µl DMF and left to activate the bromoacetic acid for 30 min, after which it was added to the resin [13]. The activated mixture was then added to the resin, and left to react for 90 min. The PNA was subsequently deprotected as described (see above) using TFA/TIS/H_2_O (95 : 2,5 : 2,5) and purified by HPLC on a 0–75% gradient (0 to 75% B in 25 min, with the compound eluting at 42% B): *m*/*z* 1376.10 observed (1375.47 calculated).

### 2.6. isoGln-GTCA-Lys-(N_3_)-NH_2_ (**4**)

In total, 5 µmol **2** was left to swell in DMF for 30 min. In a separate tube, 50 µmol DIC, HOAt and Boc-L-Glu-NH_2_ were mixed in 250 µl DMF and added without pre-activation to the resin and left to react for 90 min. The PNA was subsequently deprotected as described [3] using TFA/TIS/H_2_O (95 : 2,5 : 2,5) and purified by HPLC on a 0–75% gradient (0–75% B in 25 min, with the desired compound eluting at 38% B). *m*/*z* 1383.53 observed (1383.60 calculated).

### 2.7. isoGln-GTCA-Lys-(click)-NH_2_ (**5**)

To 50 nmol **4** was added 60 nmol MitoOct in MeOH, and the mixture was evaporated in a SpeedVac. The residue was redissolved in 200 µl MeOH and evaporated. This was repeated for five times, until completion of the reaction. The obtained product was purified by HPLC using a 0–75% gradient (0–75% B in 25 min, with the desired compound eluting at 50% B). *m*/*z* 1853.25 observed (1853.83 calculated).

### 2.8. COX8-Cys-Ac-GTCA-Lys(N_3_)-NH_2_ (**6**)

To 50 nmol **3** was added 100 nmol **1** in 60 µl 20 mM ammonium bicarbonate buffer (pH 7.5) and left to react for 1 h at 37°C. The reaction was stopped by addition of 450 µl 0.1% TFA in H_2_O and the product was purified by HPLC using a 0–75% gradient (0–75% B in 25 min, with the desired compound eluting at 53% B), which was then lyophilized and dissolved in 50 µl of millipure water. *m*/*z* 4550.33 observed (4550.45 calculated).

### 2.9. Uptake experiments in isolated rat liver mitochondria

Rat liver mitochondria were prepared by differential centrifugation as previously described [14], and diluted to a 20 mg protein ml^−1^ concentration in STE buffer (250 mM sucrose, 5 mM Tris, 1 mM EGTA, pH 7.4) and stored for a maximum of 3 h on ice. Peptide–PNA **6** was dissolved at the indicated concentrations in 100 µl KCl buffer (120 mM KCl, 10 mM HEPES, 1 mM EGTA, 1 mM ADP, 1 mM MgCl_2_, 1 mM KPi, 0.05% BSA, pH 7.4) supplemented with 10 mM potassium succinate, 4 µg ml^−1^ rotenone and 10 µM MitoOct. Where indicated carbonyl cyanide-4-(trifluoromethoxy)phenylhydrazone (FCCP, 500 nM) was added to the buffer. The buffer was warmed for 5 min at 37°C after which the reaction was initiated by addition of 5 µl mitochondria suspension (100 µg protein). The mitochondria were incubated for 1 h at 37°C, after which the reaction was stopped by the addition of 3-phenyl-1,2,4,5-tetrazine (PhTet, 50 µM). The mitochondria were pelleted by centrifugation at 16 000*g* for 1 min, and supernatant was removed. Next 100 µl 20% acetonitrile solution with 0.1% formic acid, 50 µM PhTet and 0.1 µM internal standard **5** was added to the pellet. The pellet was suspended by three cycles of freezing in solid CO_2_ (5 min) and sonication in a Grant XB2 ultrasonic bath. The obtained solution was centrifuged at 16 000*g* for 10 min. The sample was spotted onto an MALDI-plate using the bottom-layer method. Matrix (0.75 µl, 50% acetonitrile, 5 mg ml^−1^ α-cyano-4-hydroxycinnamic acid, 10 mM dibasic ammonium citrate, 0.1% TFA) was spotted on the plate and 0.75 µl sample was mixed in. The spot was left to dry at room temperature, after which another layer of 0.75 µl of matrix was added. In total, 20 spectra with 10 shots each were collected per spot, using a minimum intensity of 1000 and a maximum of 10 000 as selection criterion, averages were taken of three spots per experiment. All experiments were performed in triplicate using different mitochondrial preparations and are presented as averages ±s.e.m.

## 3. Results and discussion

### 3.1. Peptide–PNA conjugation

Previously, we have shown that click chemistry can be used to measure import of a peptide–PNA conjugate into the matrix of isolated mitochondria [9]. When carrying out these experiments, it was uncertain what, if any, type of conjugation between the MTS and the PNA would be tolerated by the translocases of the outer and inner mitochondrial membranes. We therefore chose to synthesize our targeted PNA in a linear manner on solid phase, in which the conjugate thus only consisted of amide bonds. While the synthesis of these conjugates was robust, it was time consuming and required a new complete and continuous synthesis for every conjugate that was to be tested, hampering the optimization of molecules for import. We therefore wished to examine an alternative method, which would allow for the rapid addition of an efficient peptide pre-sequence to a separately synthesized PNA by simple ligation. Addition of excess COX8-Cys-NH_2_ (**1**) to the bromo-acetylated PNA azide (**3**) in a 20 mM ammonium bicarbonate buffer rapidly drove the reaction to completion (figure 2). The product appeared as a single sharp peak in the chromatogram, separated from both peptide and PNA starting materials, allowing simple isolation of the product in good yield (80%). This procedure not only facilitates the efficient synthesis of large peptide–PNA constructs in high purity, it also allows rapid generation of libraries, and the comparison of different combinations of peptide (cell penetrating peptides, mitochondria targeting sequences, combinations thereof) and PNA (different lengths, sequences, linkers) or other types of cargo.

### 3.2. Quantification of *in organello* imported and cleaved peptide–PNA conjugate

Following the successful synthesis and purification of our peptide–PNA conjugate constructs, we wished to determine whether these molecules were imported into mitochondria, as judged both by membrane potential-dependent cleavage of the pre-sequence and formation of clicked products*.*

**Figure 3.**
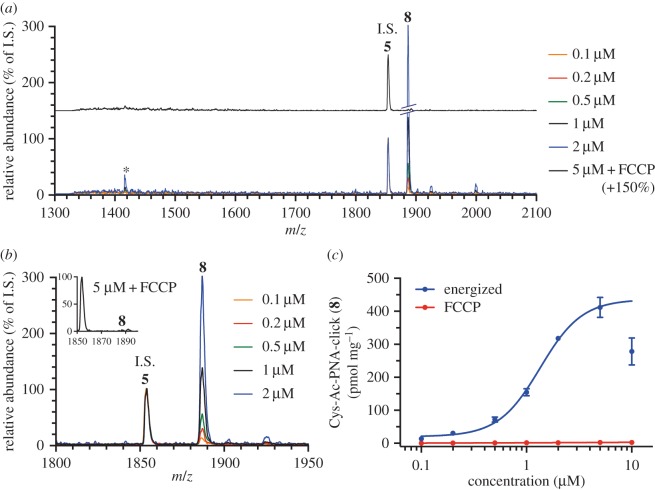
(*a*) Typical mass spectra as observed for the indicated added concentrations of **6**. Asterisk (*) indicates small amounts of cleaved but not clicked **6**, which are not observed in the presence of FCCP. (*b*) Zoom of the spectra presented in figure 3*a*. Inset: 5 µM **6** in the presence of FCCP. (*c*) The ClickIn/MPP reaction in isolated mitochondria follows a dose–response curve. All import reactions and quantifications are performed as described in the Experimental procedure section. (Online version in colour.)

Isolated rat liver mitochondria were incubated with various concentrations of peptide (0.1–10 µM) in import buffer, in the presence of MitoOct, for 1 h at 37°C. As previously described, the reaction was stopped by addition of PhTET and centrifugation. The pellet was extracted (20% acetonitrile, 0.1% formic acid, 50 µM PhTet, 0.1 μM **5**) and analysed by MALDI-TOF (figure 3 and electronic supplementary material, S1). The mass spectra indicate that the MTS was indeed removed from the clicked PNA, indicating that both MitoOct and the MPP are involved in the conversion of the peptide–PNA conjugate **6** into **8** (figure 1). Significant amounts of clicked PNA were observed in all samples compared to the FCCP control. Moreover, when FCCP was added to uncouple the mitochondrial membrane potential, no product **8** could be observed at any of the added peptide–PNA concentrations. This is consistent with FCCP not only inhibiting the ClickIn reaction, but also inhibiting the processing of COX8-PNA-click [7] by stopping import of **6**. The absence of signal in the presence of FCCP is striking (figure 3 and electronic supplementary material, S1), and enables the qualitative observation of PNA uptake in isolated mitochondria. Finally, the quantitative analysis of the reaction shows a typical dose–response curve for concentrations of **6** up to 5 µM (figure 3*c*), and indicates higher concentrations of **6** might have a toxic effect on the mitochondria, as previously observed [9]. It is interesting to note that, even though the MPP is known to cleave after the 25th residue of the COX8 pre-sequence [15], we see no evidence of the four amino acids, IHSL, predicted to remain at the N-terminal of our cleaved product. Our previous unpublished results have shown a very minor amount of the PNA product still carrying leucine at the N-terminal but not any of the remaining amino acids. It is likely that these residues are removed by a protease residing in the mitochondrial matrix.

In summary, we report the optimization of a synthesis protocol that should allow for the ligation of a peptide pre-sequence with a library of diverse PNA or other nucleobase cargoes and confirm that our methods can reliably quantify the matrix import of cleaved molecules *in organello*. We now intend to tailor these methods to investigate the possibility of mitochondrial import of such antigenomic derivatives into whole cells.

## Supplementary Material

Full spectrum of peptide-PNA ClickIn reaction with 5uM compound 6 and mass spec analysis of purified compounds
