# Supplementary material for: ClickIn: a flexible protocol for quantifying mitochondrial uptake of nucleobase derivatives
Source: Interface Focus. 2017 Apr 6;7(2):20160117. doi: 10.1098/rsfs.2016.0117 (PMC5311907; doi:10.1098/rsfs.2016.0117)
Supplement: Full spectrum of peptide-PNA ClickIn reaction with 5uM compound 6 and mass spec analysis of purified compounds [file rsfs20160117supp1.doc]

S1


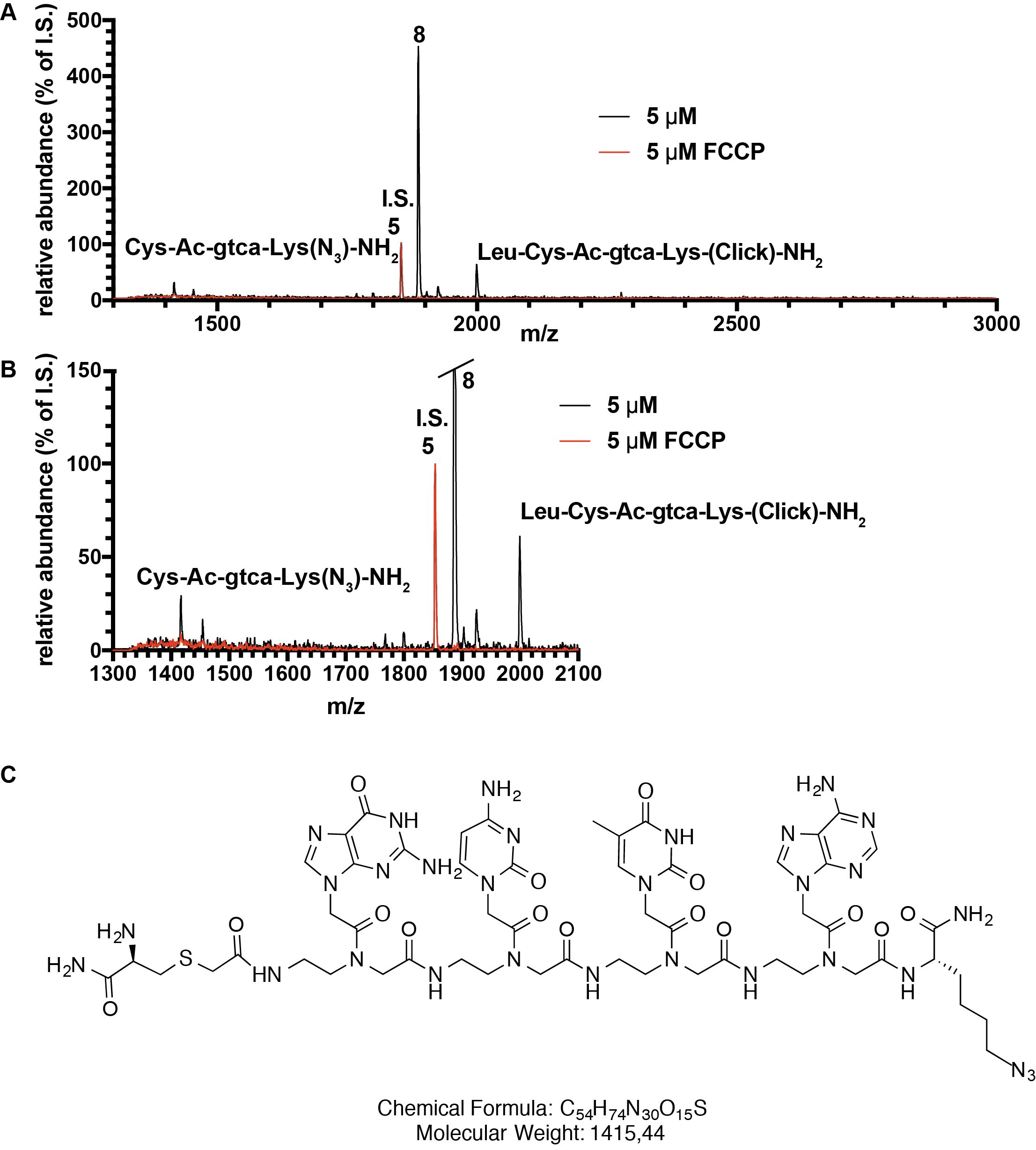


Figure S1: A. Full spectrum of peptide-PNA ClickIn reaction with 5 µM **6**. B. Zoom of S1A, which shows peaks corresponding to truncated but not clicked compound **6** only in absence of FCCP. In addition, at higher concentrations, the spectrum shows minor incomplete removal of the last leucine residue of the COX8 peptide. C. Stucture and molecular weight of the truncated unclicked compound **6**, here indicated as Cys-Ac-gtca-Lys(N_3_)-NH_2_.

Analytical HPLC was performed on a Waters Xbridge peptide BEH C18 column (300Å, 3.5µm, 4.6 mm x 250mm) using a flow rate of 1 ml/min and a gradient of 100% A (0,1% TFA in water) for 3 min, then to 100% B (0,1% TFA in ACN) in 15 min, followed by 3 min 100% B.

Figure S2: HPLC Chromatogram for compound **1**

Figure S3: HPLC Chromatogram for compound **3**

Figure S4: HPLC Chromatogram for compound **5**.

Figure S5: HPLC Chromatogram for compound **6**. Performed as described in the main article.
